# The Relationship between Exercise Intensity and Lactate Concentration on the Skin Surface

**Published:** 2009-03

**Authors:** Tetsuo Ohkuwa, Kazuhiko Tsukamoto, Kazuaki Yamai, Hiroshi Itoh, Yoshihiko Yamazaki, Takao Tsuda

**Affiliations:** 1 *Department of Material Science and Engineering, Graduate School of Engineering, Nagoya Institute of Technology, Showa-ku, Nagoya, Japan;*; 2 *Department of Computer Science and Engineering, Graduate School of Engineering, Nagoya Institute of Technology, Showa-ku, Nagoya, Japan;*; 3 *Pico-Device Co., Ltd., Offices, Incubation Center, Chikusa-ku, Nagoya, Japan*

**Keywords:** lactate, skin surface, exercise, rectus femoris, palm

## Abstract

We examined the relationship between skin surface lactate concentration on working muscle and heart rate during continuous graded cycling exercise. Sixteen healthy male volunteers participated in this study. A plastic container with 100 μl 1% ethanol was put on the skin surface on the belly of rectus femoris muscle. The workloads were 300, 600, 900 and 1080 (or 990) kpm/min, and each stage was 5 min in duration. Sample collections were performed at rest, during exercise, and recovery. The lactate concentration during exercise significantly increased compared to the basal level (p<0.05 or p<0.001). Skin surface lactate concentration was found to correlate significantly with heart rate at the exercise intensity of 360 kpm/min (r=-0.52, p<0.05), 720 kpm/min (r=-0.74, p<0.01) and 900 kpm (r=-0.53, p<0.05). This study confirmed that 1) the increase in lactate concentration on the skin surface on working muscle is associated with increase in exercise intensity (heart rate), and 2) the skin surface lactate concentration on the working muscle can be used as a parameter of exercise intensity in each subject.

## INTRODUCTION

Measuring blood lactate concentration provides information not only about changes in glycolysis (1) but also about anaerobic work capacity (2). Previous studies from our laboratory showed that peak blood lactate, which was observed after supra-maximal 400-m sprinting, correlated with running speed in both untrained subjects and long-distance runners (3). Furthermore, the peak value of blood lactate after anaerobic exercise was significantly higher in sprinters than in long-distance runners or untrained subjects (3).

Blood sampling for lactate measurement is accompanied by problems such as loss of blood, emotional stress, discomfort, and risk of infectious disease. Skin surface lactate measurement has a number of advantages compared to that of blood lactate and to the sampling materials of another non-invasive method. Although saliva and sweat collection can be performed non-invasively, the sampling of skin surface lactate is easier to collect than saliva and sweat. Moreover, there are many reports that lactate in sweat during continuous graded exercise was not associated with changes in blood lactate concentration (4, 5).

Lactate is removed by oxidation in type I fibers (6, 7) or is resynthesized to glycogen in type IIb fibers (8, 9) or to glucose in liver (10). The first lactate transporter, monocarboxylate transporter 1 (MCT-1), was highly correlated with lactate uptake in red muscle fibers (11-13), whereas monocarboxylate transporter 4 (MCT-4) is related to lactate extrusion (14, 15). The fast-twitch glycolytic white muscles, such as the white gastrocnemius muscle, expressed the most MCT-4, but MCT-1 expression was low. The slow twitch oxidative muscle, such as soleus muscle, expressed the least MCT-4 and the most MCT-1. Furthermore, MCT-1 is important for the removal of lactate from the circulation (16). Bonen *et al.* (17) reported that MCT-1, -2, -4, -7 were observed in the skin of rats. However, no other reports have been published regarding the influence of exercise on the skin surface lactate concentration.

This study investigated the influence of continuous graded cycling exercise on lactate concentration on the skin surface on rectus femoris muscle in humans.

## METHODS

### Subjects

A total of sixteen healthy male volunteers participated in this study. One subject who performed experiment I was fifty-five years old and well trained. Mean and SD (standard deviation) of age in the other fifteen subjects was 20.5 ± 1.3 years old, height was 168.8 ± 5.5 cm, weight was 59.6 ± 7.7 kg and BMI (body mass index) was 20.9 ± 2.3 kg/m^2^. These fifteen subjects performed experiment II. All subjects were in good health, claimed not to take any medicine for their health, and were non-smokers. The purpose and procedures of the study and the possible risks were fully explained to each subject before they signed an informed consent document. The study was approved by the ethical committee of the Nagoya Institute of Technology.

### Experimental protocol

All subjects had a meal at least two hours before exercise. Subjects performed a continuous graded cycling exercise (Monark-Crescent, Varburg, Sweden). The workloads were 300 (1.0 kp), 600 (2.0 kp), 900 (2.5 kp), and 990 (2.75 kp) or 1080 (3.0 kp) kpm/min, and each stage was 5 min in duration. A pedaling frequency of 60 rpm was maintained constant through the exercise duration using the metronome.

All experiments were performed after the subjects rested for 20 min. The room temperature was set at 26°C. Experiment I, the skin surface lactate of rectus femoris muscle was collected ten times from one subject. In experiment II, fifteen subjects performed incremental cycle exercise at four different intensities for 20 min. The skin surface lactates on rectus femoris muscle and on palm of left hand were collected at rest, during exercise and recovery.

The method of collection of lactate present on the skin surface was as follows. The skin surface was cleaned with 1% ethanol. It is very important to clean the skin before the lactate collection. 100 μl of ethanol (1% ethanol-water) was put into the sampling collector (cylindrical plastic container, 7.2 mm in diameter and 6.6 mm in height). The plastic container were put on the skin surface on the belly of the rectus femoris muscle and the palm (the root of thumb). The ethanol solution came in contact with the skin surface for 60 sec. We waited exactly for 4 min, and a cylindrical plastic container was put on the skin surface on the rectus femoris muscle and the palm of left hand to get skin surface lactate concentration at the resting condition. The lactate concentrations measured twice at rest. The lactate concentration of the first sample was very high. Accordingly, we used the value of the second measurement as the basal level of skin surface lactate. After the second sample collection at rest, the cycle exercise started. Sample collections were performed 4-5 min and 9-10 min after cleaning the skin surface at rest, and 4-5, 9-10, 14-15, 19-20 min during exercise, and 4-5, 9-10, 14-15 min after exercise. The exercise stopped for 6-7 sec before and after sampling. Each stage was 5 min in duration. The sampling on skin surface was performed in the same place during experiments.

### Determination of lactate concentration on skin surface

The samples were quantitatively measured by HPLC with a UV detector. The HPLC apparatus consisted of a UV detector (SPD-20A, Shimadzu, Kyoto, Japan), an LC-20AD (Shimadzu, Kyoto, Japan), a Chromatocorder 21 (SCI, Tokyo, Japan), and a column (CAPCELL PAK C18 UG120S-5; 4.6 mm × 150 mm, Shiseido, Kyoto, Japan). The mobile phase was 7.3 mM phosphoric acid and 88 mM sodium dihydrogenphosphate solution. Injection volume was 20 μl and flow rate was 0.5 ml/min. The wavelength was 210 nm. The quantification of lactate was carried out by comparison of each peak area with that of the corresponding standard. All measurements were carried out in duplicate. The heart rates were continuously measured at rest, during exercise and after exercise by a heart rate monitor (C810i, Polar Electronic, Kemperle, Finland).

### Statistical analysis

Values were expressed as mean and standard errors of the mean (M ± SEM). Data were analyzed using a one-way analysis of variance (ANOVA) with p<0.05 determined to be statistically significant. When differences were obtained, post hoc analysis was performed using Fisher’s PLSD. Statistical analysis was calculated with Stat View (v. 5.0, Avaccus Concepts).

## RESULTS

### Experiment I

**Figure 1:**
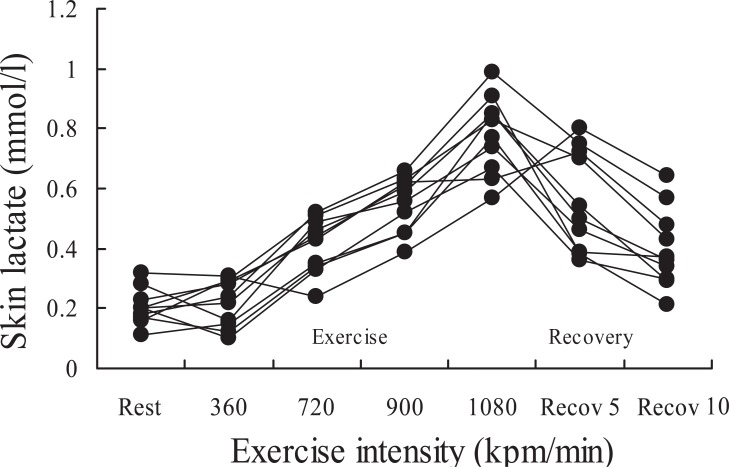
Lactate concentration of skin surface on working muscle at rest, during cycle exercise and recovery when one subjects performed the same exercise ten times.

**Figure 2:**
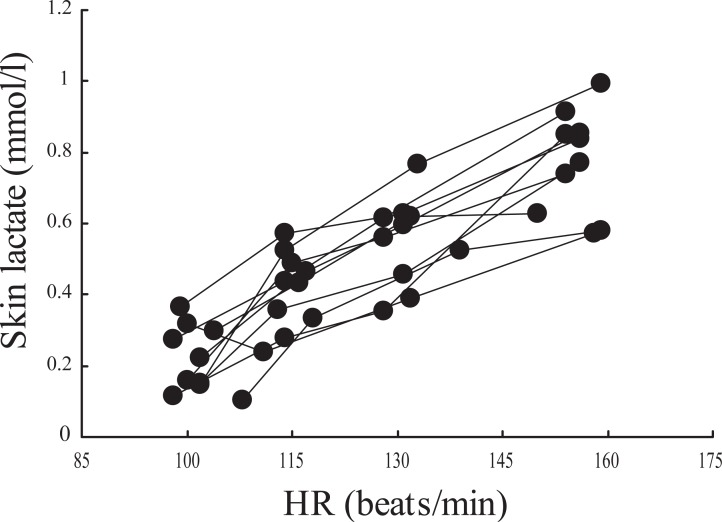
Relationship between hear rate and skin lactate concentration on working muscle during exercise. One subject performed the same exercise ten times.

Figure 1 demonstrates changes in lactate concentration on the skin surface at rest, during exercise, and after exercise when one subject performed the same exercise ten times. Figure 2 demonstrates the relationship between skin surface lactate concentration and heart rate when one subject performed the same exercise ten times. Skin surface lactate concentration on working muscle increased with increased exercise intensity and heart rate.

### Experiment II

**Figure 3:**
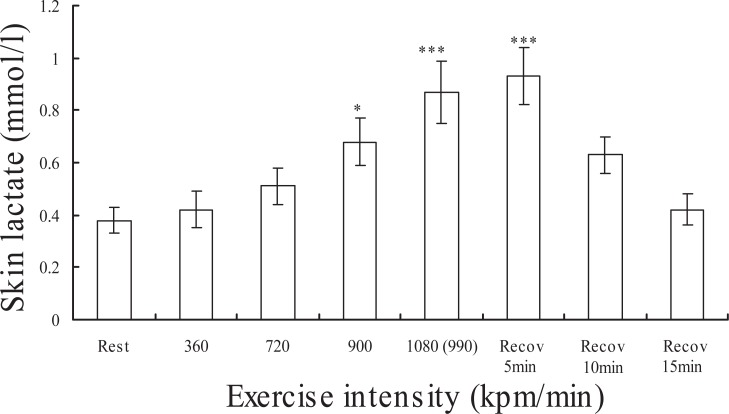
Changes in lactate concentration of skin surface on working muscle at rest, exercise and recovery in fifteen subjects. Values are mean±SEM. ***p<0.001 significant difference compared to basal level. *p<0.05 significant difference compared to basal level.

**Figure 4:**
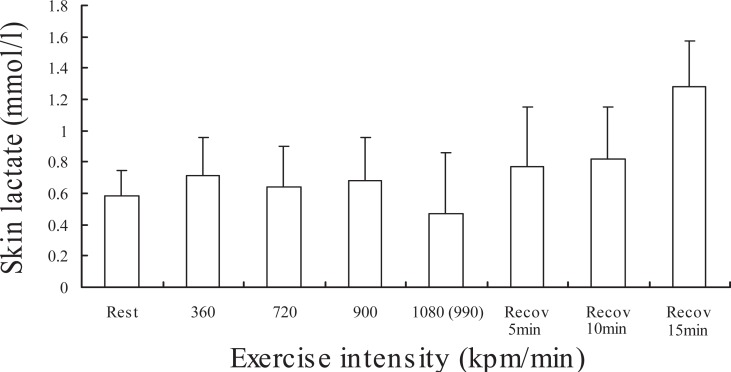
Changes in lactate concentration of skin surface on palm (the root of thumb) at rest, exercise and recovery in fifteen subjects. Values are mean ± SEM.

**Figure 5:**
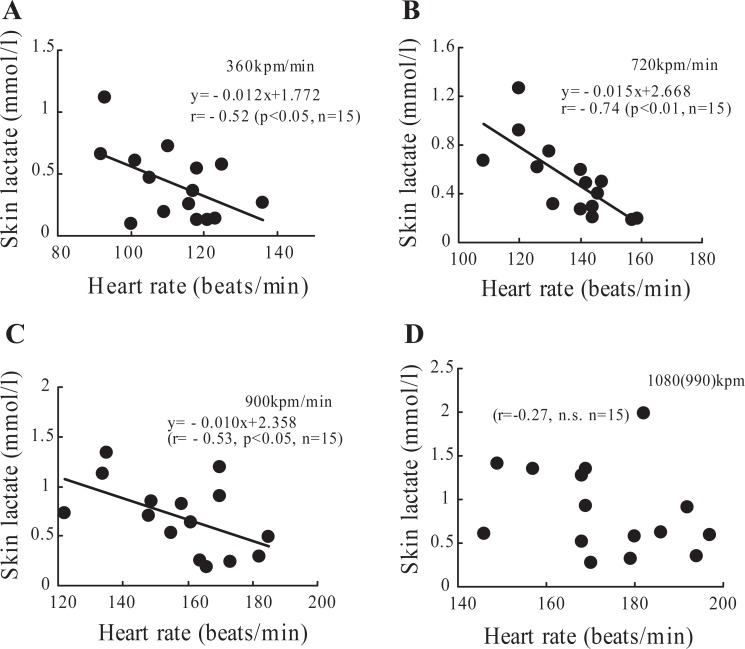
Relationship between heart rate and skin surface lactate on working muscle at 360kpm/min (A), 720kpm/min (B), 900kpm/min (C) and 1080 (990)kpm/min (D) in fifteen subjects.

Figure 3 demonstrates changes in lactate concentration on the skin surface at rest, during exercise, and after exercise in fifteen subjects. Though the lactate concentration at the low intensities (360 and 720 kpm) did not change compared to the basal level, significantly increased at the intensity of 900 and 1080 (990) kpm and 5 min after exercise (p<0.05 or p<0.001). Figure 4 demonstrates changes in lactate concentration on the palm of on the left hand at rest, during exercise, and after exercise in fifteen subjects. The lactate concentration in the surface on palm during exercise and recovery did not change compared to the basal level. The relationships between skin surface lactate concentration on the working muscle and heart rate at 360, 720, 900 and 990 (1080) kpm/min are shown in Figure 5. Skin surface lactate concentration on working muscle was found to correlate significantly with heart rate at the exercise intensity of 360 kpm/min (r=-0.52, p<0.05), 720 kpm/min (r=-0.74, p<0.01) and 900 kpm (r=-0.53, p<0.05). There are no significant correlations at the intensity of 990 (1080) kpm/min.

## DISCUSSION

In experiment I, lactate concentration on the skin surface on working muscle increased with exercise intensity and heart rate when lactate of skin surface was measured ten times in one subject (Figs. 1 and 2). In the experiment II, lactate concentration on the skin surface on rectus femoris muscle increased with increase in exercise intensity in fifteen subjects (Fig. 3). In some subjects, the lactate concentration on the skin surface on working muscle during exercise increased remarkably while in others it increased only a little. McCullagh *et al.* (12) reported that chronic muscle contraction increases MCT-1 concentrations in both red and white skeletal muscles. It has been reported that there is a strong positive relationship between MCT-1 and lactate uptake (11-13), and the researchers hypothesize that MCT-1 is the lactate entry point into the muscle cell. It has been suggested that MCT-1 may be mainly involved in lactate uptake into the muscle, while MCT-4 is involved with extruding lactate (14, 15). Bonen *et al.* (17) reported that MCT-1, MCT-2, MCT-4 and MCT-7 were observed in rat skin. Though this report deals with data on rat skin, it speculated that these MCTs are also present in human skin. In this study, the increase in lactate concentration on the skin surface on working muscle during exercise may be due to the presence of MCTs.

There was a negative correlation between lactate concentration on the skin surface on rectus femoris muscle and heart rate at the exercise intensity of 360 kpm/min, 720 kpm/min and 900 kpm/min, suggesting that lactate concentration on the skin surface on working muscle may be correlated with heart rate (Fig. 5). Rate of increase in skin lactate concentration of subjects with lower heart rate was higher than with higher heart rate at the same exercise intensity. Pilegaard *et al* (18) demonstrated in humans that dynamic knee extensor exercise training increased in MCT-1 and MCT-4 in vastus lateralis muscle. Dubochaud *et al.* (19) also reported that leg cycle endurance training increased the MCT-1 and MCT-4 in human vastus lateralis muscle. In rat experiments, McCullagh *et al.* (12) and Baker *et al* (20) reported MCT-1 protein expression was increased after chronic electrical muscle stimulation or treadmill running training in the red and white muscles.

On the other hand, the lactate concentration on the skin surface on palm unchanged during exercise and recovery (Fig. 4). This result indicates that exercise did not change the skin lactate concentration on the non-working muscle.

This study confirmed that the increase in lactate concentration on the skin surface on working muscle is associated with an increase in exercise intensity (heart rate), and the skin surface lactate concentration on the working muscle can be used as a parameter of exercise intensity in each subject.
